# Related Entropy Theories Application in Condition Monitoring of Rotating Machineries

**DOI:** 10.3390/e21111061

**Published:** 2019-10-29

**Authors:** Liansheng Liu, Zhuo Zhi, Hanxing Zhang, Qing Guo, Yu Peng, Datong Liu

**Affiliations:** School of Electronics and Information Engineering, Harbin Institute of Technology, Harbin 150080, China; lianshengliu@hit.edu.cn (L.L.); zhuozhi@hit.edu.cn (Z.Z.); zhanghx@hit.edu.cn (H.Z.); qguo@hit.edu.cn (Q.G.); pengyu@hit.edu.cn (Y.P.)

**Keywords:** entropy theory, feature extraction, fault detection, fault diagnosis, fault prognostics, rotating machinery

## Abstract

Rotating machinery plays an important role in various kinds of industrial engineering. How to assess their conditions is a key problem for operating safety and condition-based maintenance. The potential anomaly, fault and failure information can be obtained by analyzing the collected condition monitoring data of the previously deployed sensors in rotating machinery. Among the available methods of analyzing sensors data, entropy and its variants can provide quantitative information contained in these sensing data. For implementing fault detection, diagnosis, and prognostics, this information can be utilized for feature extraction and selecting appropriate training data for machine learning methods. This article aims to review the related entropy theories which have been applied for condition monitoring of rotating machinery. This review consists of typical entropy theories presentation, application, summary, and discussion.

## 1. Introduction

**Figure 1:**
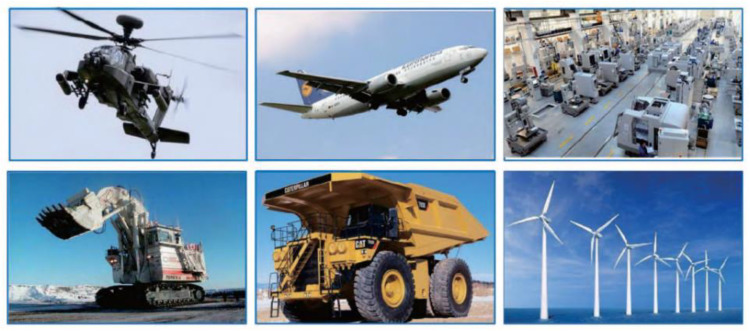
Typical applications of the rotating machinery [1].

Rotating machinery has been applied in many practical industry applications, such as helicopters, civil aircraft, machine centers, tracked loaders, mining vehicles, and wind turbines, as shown in Figure 1 [1].

In general, rotating machinery work under the conditions of harsh environment and long-time operation. It is inevitable to face various kinds of faults in rotating machinery, such as crack, pitting, wear, and break [2,3,4]. These faults may bring the breakdown of the system, cause lots of cost and even catastrophic accidents. Therefore, researchers from industry and academia fields have paid much attention to implement the fault detection, diagnosis, and prognostics of rotating machineries [5,6,7,8]. Especially, the variable speed of rotating machinery brings big challenges to realize these objectives [9]. If the condition monitoring data are not enough, the accelerated degradation model can be utilized [10,11]. The localization diagnosis of the defectiveness is also worth studying [12]. Many methods have been proposed to achieve accurate and stable results of condition monitoring.

Traditionally, these methods include stochastic statistics [13,14], Bayesian inference [15], and signal processing [16,17,18]. With the development of machine learning, some intelligent methods have been proposed to achieve better performance of fault detection, diagnosis, and prognostics. The typical methods in this category include the regrouping particle optimization [19], the step-by-step fuzzy diagnosis [20], the adaptive density peaks search [21], the combination of statistic filter and wavelet package transform [22], and the fusion of dynamic Bayesian networks and Monte Carlo algorithm [23]. Recently, the method based on deep learning has drawn the attention of researchers [24]. Some studies in this area include the deep learning framework using the improved logistic Sigmoid and transfer learning [25,26], the convolutional neural network-based hidden Markov model [27], the adaptive learning rate deep belief network combined with Nesterov momentum [28], the combination of sparse autoencoder and deep belief network [29].

**Figure 2:**
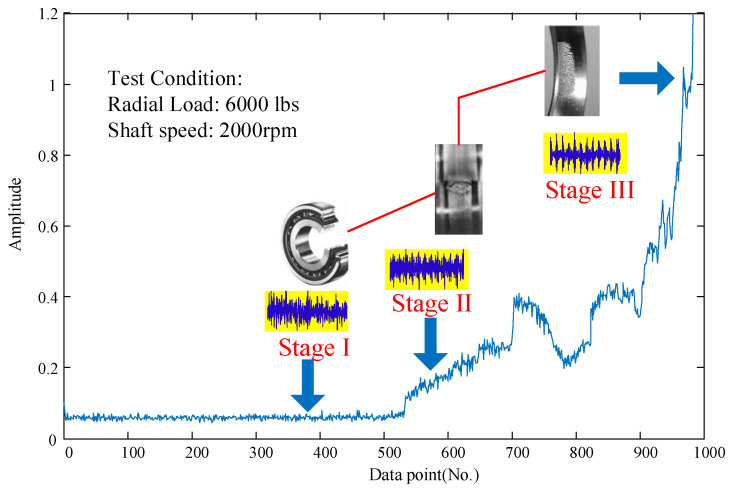
The whole life condition of a real bearing [30].

The existing methods indeed provide feasible ways for implementing condition monitoring of rotating machinery. In the practical application, the whole life of rotating machinery (e.g., a bearing) consists of three stages, as shown in Figure 2. Stage I, stage II, and stage III are normal operation, early fault, and failure, respectively [30].

During the whole life of rotating machinery, vibration data are often utilized as the input of the appropriate methods to carry out condition assessment. There are also some other kinds of condition monitoring data (e.g., temperature, oil analysis, and acoustics emission), which can be used to achieve the condition estimation of rotating machinery. How to improve the performance of the utilized methods is paid much attention from industrial and academic areas. One research branch is to select the appropriate data for assessing the condition of rotating machinery. If the condition information contained in the monitoring data can be determined quantitatively, it may bring positive effect on the condition monitoring assessment results.

As a statistical measure metric, Shannon entropy can provide the dynamic change and complexity information of the data series [31]. At present, related entropy theories have been applied for fault detection, diagnosis, and prognostics of rotating machineries [32,33,34,35,36]. The related entropy theories include Shannon entropy, spectral entropy, wavelet entropy, Rényi entropy, permutation entropy, sample entropy, approximate entropy, fuzzy entropy, etc. In this way, the condition monitoring data of rotating machinery can be achieved quantitative information analysis. Then, the condition of rotating machinery can be assessed by the fusion of this quantitative information and appropriate methods. This article aims to provide a comprehensive review of related studies of condition monitoring of rotating machinery using entropy theories. Different from the existing review [1], the objects of this review focus on bearing and gear. Other typical rotating machineries are also reviewed. The related studies are reviewed in detail, which are expected to provide a valuable reference for implementing condition monitoring of rotating machinery.

The rest of this article is organized as follows. Section 2 introduces related entropy theories. Section 3, Section 4 and Section 5 reviews the applications of entropy theories on condition monitoring of bearing, gear, and other rotating machinery. Section 6 gives a case study. Section 7 draws the summary and describes some potential applications.

## 2. Shannon Entropy and Its Variants

In this section, the basic definition of Shannon entropy is introduced. Then, the variants of Shannon entropy are presented, which are basic theories for carrying out condition monitoring of rotating machinery.

### 2.1. Shannon Entropy

The definition of entropy is proposed by Shannon [37]. For the discrete data series $X=\left\{x_{1},x_{2},...,x_{n}\right\}$ with *n* different values of *x_i_ (i = 1*, *2*, …, *n)*, its Shannon entropy is defined by

(1) $$H(X)=-\sum_{i=1}^{n}p\left(x_{i}\right)\log\left(p\left(x_{i}\right)\right),$$

where *p* is the ratio of $\left\{x_{i}\right\}$ number over the total number of data, and *n* is the number of different data. 

For a continuous random variable *X* with the probability density of *f*(*x*), its Shannon entropy is defined by

(2) $$H(X)=-\int_{S}f\left(x\right)\log f\left(x\right)dx,$$

where *S* denotes all random variables.

In the above two equations, if the base of the logarithm is *b*, the entropy can be denoted as *H_b_*(*X*). Two kinds of entropy are usually utilized, and bases are 2 and *e*, respectively. If the base is set to 2, entropy is expressed in bits. If the base is set to *e*, entropy is expressed in nats. The convention is that 0log0 = 0. It is easily justified by continuity since *x*log*x*→0 as *x*→0. Adding terms of zero probability does not change the entropy.

In fact, entropy refers to the uncertainty of a single random variable. The more complex the random variable is, the larger the entropy value is. To help understand the definition of entropy, we take a random variable as an example, which has a uniform distribution over 16 outcomes. The entropy of this random variable is

(3) $$H\left(X\right)=-\sum_{i=1}^{16}p(i)\log p(i)=-\sum_{i=1}^{16}\frac{1}{16}\log\frac{1}{16}=\log16=4\;\mathrm{bit}s,$$

which agrees with the number of bits needed to describe *X*.

### 2.2. Variants of Shannon Entropy

#### 2.2.1. Energy Entropy

Energy entropy can be used to quantify the regularity of the data series. For analyzing condition monitoring data of rotating machinery, the intrinsic mode function (IMF) is usually used. In the following analysis, IMF is taken as an example. If there are *n* IMFs, the corresponding energy entropy is calculated as follows [38].

(1) The energy of the *i*th IMF is calculated by

(4) $$E_{i}=\sum_{j=1}^{m}{\left|c_{ij}\right|}^{2},$$

where *m* refers to the length of IMF.

(2) For *n* IMFs, the total energy can be calculated by

(5) $$E=\sum_{i=1}^{n}E_{i}.$$

(3) Based on the above two steps, the energy entropy of these IMFs can be achieved by

(6) $$E_{en}=-\sum_{j=1}^{n}p_{i}\log(p_{i}),$$

where $E_{en}$ indicates the energy entropy of the data series and $p_{i}=E_{i}/E$ denotes the ratio of the *i*th IMF energy to the total energy entropy.

#### 2.2.2. Permutation Entropy

Permutation entropy is proposed to measure the complexity of data series [39]. In which, condition monitoring data are first described through the phase space reconstruction. The specific value of permutation entropy can reflect the dynamic features of the monitored machinery.

Let $\left\{x{\left(t\right)}_{t=1,2,\cdots ,T}\right\}$ denote the condition data series. All n! permutation π of order *n* which means as possible order types of *n* different numbers. For each π, the relative frequency is defined by

(7) $$p(\pi )=\frac{num\left\{t|0\leq t\leq T-n,(x_{t+1},\cdots ,x_{t+n})has\;type\;\pi \right\}}{T-n+1}.$$

The permutation entropy with n≥2 can be achieved by

(8) H(n)=−∑p(π)logp(π).

Permutation entropy can measure the complexity of the chaotic data series. It is still effective when there is dynamical and observational noise. Its main advantages include high calculation efficiency and robustness to noise.

However, permutation entropy cannot classify the defined patterns of the particular design well [40]. To solve this problem, Zheng et al. [41] propose the generalized composite multiscale permutation entropy (GCMPE), which is illustrated as follows.

For the data series {x(i),i=1,2,…,N}, the coarse-grained data series $\left\{y_{k}^{\left(\tau \right)}\right\}$ can be expressed by

(9) $$y_{k}^{\left(\tau \right)}=\frac{1}{\tau }\sum_{i=(j-1)\tau +k}^{j\tau +k-1}(x_{i}-{\bar{x}}_{i}),\;1\leq j\leq N/\tau ,\;2\leq j\leq \tau .$$

where τ is the scale factor, and k is the index of the generalized coarse-grained series $y_{k}^{\left(\tau \right)}\left(k=2,\ldots ,\tau \right)$. ${\bar{x}}_{i}=\frac{1}{\tau }\sum_{k=0}^{\tau -1}x_{i+k}$ is the mean of the original τ data.

Finally, the generalized composite multiscale permutation entropy can be obtained by

(10) $$GCMPE(X,\tau ,m,\lambda )=\frac{1}{\tau }\sum_{k=1}^{\tau }PE(y_{k}^{\left(\tau \right)},m,\lambda ).$$

where m refers to the embedding dimension, and λ denotes the time delay.

#### 2.2.3. Rényi Entropy

Rényi entropy is proposed to quantitatively measure the irregularity, uncertainty, and randomness of data series. Its definition is given by [42]

(11) $$REN_{\alpha }(X)=-\frac{\alpha }{1-\alpha }\sum \log_{2}p_{i}^{\alpha },$$

where $p_{i}$ denotes the probability of $\left\{x_{1},x_{2},...,x_{n}\right\}$ and α is the order. It requires that α cannot be 1. For α≥2, Rényi entropy can provide the lower bound of the corresponding smooth entropy, which is a measure for the number of uniformly random bits [43].

The advantages of Rényi entropy can be summarized in two terms. The first is that it is changed with the additive constant. In this way, it can reflect the rescaling of the data series. The second is that it is not changed under the condition of different density functions. 

#### 2.2.4. Sample Entropy

Sample entropy is proposed to achieve the complexity estimation of data series [44]. It mainly measures the complexity from the perspectives of embedding dimension *m* and similarity coefficient *r*. A larger value of sample entropy indicates a more complex data series. On the other hand, a smaller value of sample entropy indicates smaller complex. Sample entropy is defined by

(12) $$SampEn=-\ln(\frac{B^{m+1}(r)}{B^{m}(r)}),$$

where $B^{m}(r)$ refers to the mean value of pattern mean count, as given by

(13) $$B^{m}(r)=\frac{1}{N-m+1}\sum_{i=1}^{N-m+1}\left[\frac{1}{N-M}num\{d[x(i),x(j)]<r\}\right]i=1,2,\cdots ,N-m+1,\;i\neq j,$$

where r is the tolerance factor, m refers to the embedding dimension, and N is the length of the data series. num{d[x(i),x(j)]<r} denotes the count of the distance between x(i) and x(j) which is lower than r. If i is not equal with j, it means that sample entropy does not contain self-matches. For the practical application, it is better to choose m=2 and r=(0.1~0.25)∗SD, in which SD refers to the standard deviation [45]. To solve this problem, Zhang et al. [46] propose the multiscale sample entropy, which can provide the complexity of the original data series over a range of scales. Let $\left\{x_{1},x_{2},\ldots ,x_{N}\right\}$ be data series and i be the scale for realizing the coarse-graining. Several coarse-grained data series $\left\{y^{\left(\tau \right)}\right\}$ can be achieved by

(14) $$y_{j}^{\left(\tau \right)}=\frac{1}{\tau }\sum_{i=(j-1)\tau +1}^{j\tau }x_{i},\;1\leq j\leq N/\tau ,$$

where τ denotes the scale factor. It should be defined as a positive integer. If τ is set to be 1, $\left\{y^{\left(1\right)}\right\}$ refers to the original data series.

Based on each sample entropy, the multiscale sample entropy can be realized by

(15) $$MSE(x,\tau ,m,r)=SampEn(y_{j}^{\left(\tau \right)},m,r).$$

By utilizing multiscale sample entropy, the dynamical features of the data series can be mined adequately. However, there is an aliasing problem when multiscale sample entropy is utilized. Besides, if the scale factor is increased, the corresponding standard deviation will be lower.

#### 2.2.5. Approximate Entropy

To quantitatively describe the irregularity and unpredictability of data series, approximate entropy is proposed [47]. Its definition is given by

(16) $$ApEn=\phi^{m}(r)-\phi^{m+1}(r),$$

where r refers to the tolerance, and m denotes the pattern length. $\phi^{m}(r)$ can be calculated by

(17) $$\phi^{m}(r)=\frac{1}{N-m+1}\times \sum_{i=1}^{N-m+1}\ln\left[\frac{1}{N-m+1}num\left\{d\left[x\left(i\right),x\left(j\right)\right]<r\right\}\right],$$

where *N* refers to the length of the data series and num{d[x(i),x(j)]<r} indicates the count of the distance between x(i) and x(j), which is lower than r. The distance denotes the maximum absolute difference of the corresponding scalar components. In fact, the approximate entropy is the negative average of the conditional probability. 

The advantages of approximate entropy can be summarized in three terms. The first is that it has the anti-interference feature. The second is that it can realize the stable prediction result in the condition of the short data series. The last is that it can be applied to analyzing the random signals and certain signals. However, approximate entropy has some deficiencies, such as the low calculation efficiency and dependence on the length of the data series.

#### 2.2.6. Fuzzy Entropy

Fuzzy entropy is proposed to improve the boundary of two classes [48]. The innovative idea is to replace the Heaviside function in the sample entropy by Gaussian function. Let {x(i),i=1,2,⋯,N} be data series, the similarity of fuzzy entropy is defined by

(18) $$D_{ij}^{m}=\mu (d_{ij}^{m},n,r)=e^{-\ln2{\left(d_{ij}^{m}/r\right)}^{n}},$$

where r refers to the similarity tolerance. The distance between $X_{i}^{m}$ and $X_{j}^{m}$ is represented by $d_{ij}^{m}$. To achieve fuzzy entropy, the function $\varphi^{m}$ is defined by

(19) $$\varphi^{m}(n,r)=\frac{1}{N-m}\sum_{i-1}^{N-m}\left(\frac{1}{N-m-1}\sum_{j=1,j\neq i}^{N-m}D_{ij}^{m}\right).$$

Based on the above equation, fuzzy entropy can be realized by

(20) $$FE(m,n,r,N)=\ln\varphi^{m}(n,r)-\ln\varphi^{m+1}(n,r).$$

Fuzzy entropy can achieve better performance on anti-interference because it is insensitive to the background noise. However, the calculation efficiency of fuzzy entropy is very low. The biggest problem is that it may generate unreliable results. To solve this problem, multiscale fuzzy entropy is proposed [49]. For the data series $\left\{X_{i}\}=\{X_{1},X_{2},\cdots ,X_{N}\right\}$, the coarse-grained series $y_{j}^{\tau }$ can be obtained by

(21) $$y_{j}^{\tau }=\frac{1}{\tau }\sum_{i=(j-1)\tau +1}^{j\tau }x_{i},1\leq j\leq \frac{N}{\tau },$$

where τ=1,2,…,N refers to the positive integer.

Then, multiscale fuzzy entropy with the function of scale factor τ can be realized by

(22) $$MFE(x,\tau ,m,n,r)=FuzzyEn(y_{j}^{\tau },m,n,r).$$

In the following analysis, the implemented condition monitoring studies of bearing, gear, and other typical rotating machinery is based on the aforementioned theories. The typical methodologies are illustrated in terms of the utilized entropy theory and condition monitoring approach.

## 3. Condition Monitoring of Bearing

**Figure 3:**
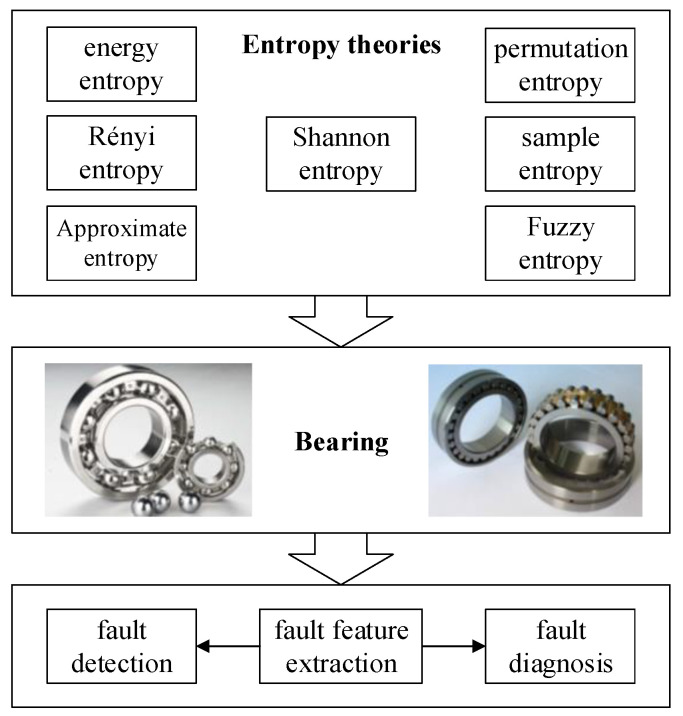
Related entropy theories applications in condition monitoring of bearing.

Bearing is the most widely utilized rotating machinery. Its condition monitoring has drawn much attention from the industry and academia field. This section illustrates the related works which have utilized entropy theories to achieve condition monitoring of bearing, as shown in Figure 3.

### 3.1. Application of Shannon Entropy on Bearing

As an effective metric of uncertainty, Shannon entropy can analyze the fault information contained in the monitoring data of bearing, as illustrated in Table 1. Vibration signal is usually utilized for implementing condition monitoring of rotating machinery. The achieved information by Shannon entropy can be used as the input of appropriate methods (e.g., support vector machine, learning vector quantization and singular value decomposition) [50,51]. 

Compared with the traditional vibration signal, the acoustic emission signal can reflect the defect size and location. The combination of wavelet packet transformation and Shannon entropy can analyze the original acoustic emission signal effectively [52]. To be specific, the method consists of four steps, including statistical analysis, fault diagnostics, defect size calculation, and prognostics. By identifying the trend of acoustic emission signal, the fault development can be predicted in advance. In addition, a method based on empirical mode decomposition and entropy is proposed to monitor the working condition of bearing [53]. The modified z-score is implemented and utilized to detect changes of features of Shannon entropy, which is applied to detecting fault by analyzing two bearing datasets [54].

### 3.2. Application of Energy Entropy on Bearing

The typical application of energy entropy in the condition monitoring of bearing is summarized in Table 2. In fact, energy entropy is usually utilized with the intrinsic mode function. It can effectively measure the valuable information contained in the intrinsic mode function. Empirical mode decomposition can self-adaptively decompose the complicated signal into different intrinsic mode functions. Therefore, energy entropy has been combined with empirical mode decomposition in many works, as given in [55]. To solve the problem of empirical mode decomposition, the ensemble empirical mode decomposition can be used [38].

Another typical study is that the fusion of wavelet transform and energy entropy is applied to achieving condition assessment of bearing [56]. Besides the general fault, weak fault and health degradation can be realized using the customized standard multiwavelets. The potential basis functions for weak fault are formulated and the influence of the variable working condition is overcome. Mahalanobis distance is employed to monitor health conditions and track performance degradation. The Teager energy entropy ratio gram is proposed to accurately identify the resonant frequency band under strong background noise, which takes the wavelet packet transform as the signal frequency band decomposition method [57]. Similar to this method, Yao et al. [58] use wavelet packet energy entropy and the local outlier factor algorithm for real-time chatter detection and suppression of intelligent spindle. In addition, the energy of Shannon entropy and local decomposition can be used to mine the condition monitoring data of bearing [59,60,61]. By analyzing these features with machine learning methods, the result of condition assessment can be enhanced to a large degree. A novel evaluation index defined as characteristic frequency band energy entropy is proposed to extract the defective features of rotors. The fault type can be automatically identified when it is combined with support vector machine [62]. Entropy theory can also be combined with deep learning method for bearing fault diagnosis. In [63], a multi-step progressive diagnosis method based on energy entropy theory and hybrid ensemble auto-encoder is proposed to realize the condition assessment of bearing.

### 3.3. Application of Permutation Entropy on Bearing

The related works of permutation entropy application on bearing condition monitoring are given in Table 3. As a metric of non-linear behavior measurement, permutation entropy has been utilized to realize condition monitoring data analysis. The most widely used strategy is the combination of permutation entropy and decomposition methods, as given in [64,65,66,67,68,69,70]. Similar to those works in the category of energy entropy, these achieved features are mainly the metrics in time domain, frequency domain, and their combination. To realize condition assessment, it needs to utilize some data analysis methods, such as support vector machine and extreme learning machine.

One typical method is that the wavelet packet transform and decomposition are combined with permutation entropy to enhance the ability of feature extraction [71,72]. The method based on wavelet analysis is effective to extract features contained in the weak transient signal. Similarly, variation mode decomposition is also used to recognize fault of bearing combined with permutation entropy [73,74]. These methods are not only capable of extracting accurately fault features but also can distinguish availably multi-class fault patterns. In addition, Yasir et al. [75] propose a method based on data decomposition technique and multi-scale permutation entropy which attaches reliable and damage-sensitive effects. Specifically, the local mean decomposition is utilized to decompose the vibration data or acceleration measurement into separate functions, and multi-scale permutation entropy is calculated to extract nonlinear features. A method based on permutation entropy and manifold-based dynamic time warping is proposed in which permutation entropy and manifold-based dynamic time warping are used to effectively diagnose bearing faults under variable working conditions and fault severity [76]. Lv et al. [77] propose an adaptive local iterative filter decomposition method based on permutation entropy, which solves some problems of adaptive local iterative filtering about the selection of threshold parameters and the number of components.

One kind of relatively easy method is based on the fusion of permutation entropy and support vector machine [78], which usually includes two steps. The first step is to achieve dynamic features using permutation entropy. Then, these features are processed by support vector machine to realize fault diagnosis. In fact, the support vector machine is one kind of classifier to identify normal and fault conditions. There are also some other types of support vector machines combined with permutation entropy-based method for fault diagnosis. Xu et al. [79] combine compound multiscale permutation entropy with support vector machine and particle swarm optimization for bearing fault diagnosis. A method based on improved multiscale permutation entropy, laplacian score, and least squares support vector machine-quantum behaved particle swarm optimization is proposed in [80]. Similar to this idea, Huo et al. [81] propose a method by integrating the fine-to-coarse multiscale permutation entropy, laplacian score and support vector machine. Multiscale permutation entropy is combined with improved support vector machine based on binary tree for bearing vibration feature extraction, as given in [82]. To overcome the problem that the coarse-grained data series in multiscale permutation entropy becomes shorter with the increase of the scale factor, the time-shift multi-scale weighted permutation entropy approach is proposed which can achieve high recognition rate on bearing fault diagnosis when combined with gray wolf optimized support vector machine [83]. Ensemble empirical mode decomposition is combined with weighted permutation entropy and an improved support vector machine ensemble classifier for bearing fault recognition [84]. The adaptive neuro-fuzzy classifier is used to distinguish normal and fault features obtained by permutation entropy [85]. The work in [86] takes advantage of high-dimensional space tends to reveal the dynamic behavior of the original data. Then, the bearing faults can be identified by using these features. Feature space reconstruction is utilized with multiscale permutation entropy to distinguish different fault categories of severity of rolling bearing [87]. In [88], the composite multi-scale weighted permutation entropy and extreme learning machine are combined for fault diagnosis of bearing. Xue et al. [89] propose a two-step fault diagnosis scheme based on statistical classification and random forests-based classification.

### 3.4. Application of Rényi Entropy on Bearing

The typical works, which have utilized Rényi entropy to realize condition assessment of bearing, are given in Table 4. In [90], authors employ Rényi entropy to extract fault features of bearing and the output of the Gaussian process model is used as a likelihood distribution. This work proves that the progressing fault implicates raising dissimilarity in the distribution of energy across the vibrational spectral band sensitive to the bearing fault. With statistical moments widely used for condition monitoring and diagnosis of bearing, the series of new diagnostic indices are originated from Rényi entropy to demonstrate vibration characteristics [91]. It can be considered as a generalization of the traditional statistical method. Singh et al. [92] use an ensemble empirical mode decomposition and Jensen Rényi divergence-based methodology to evaluate the degradation of bearing.

### 3.5. Application of Sample Entropy on Bearing

The typical studies of sample entropy on condition monitoring of bearing are summarized in Table 5. As an ideal source of intelligent fault diagnosis for complex machines, acoustic signals have inherent properties that are non-directional and insensitive to structural resonance. Singular value decomposition and sample entropy can be used to extract fault characteristics due to their sensitivity to irregular and periodic fault signal [93]. Zhang et al. [94] propose a novel scheme for bearing fault diagnosis based on lifting wavelet packet transform and sample entropy. Bearing vibration signals are decomposed into different frequency sub-bands through lifting wavelet packet transform. The sample entropy values of all components are calculated as original features to characterize the complexity of bearing vibration signals within corresponding frequency bands. Seera et al. [95] propose a hybrid intelligent model consisted of the fuzzy min-max neural network and the random forest model, in which the power spectrum and sample entropy features are used for fault classification. Local mean decomposition is combined with sample entropy and energy ratio for fault diagnosis that local mean decomposition has the characteristics of self-adaptive time-frequency. In this way, it can be used for analyzing complex signals. The regularity and characteristics of vibration signals can be achieved, as given in [96]. To solve the problem that the sound signal of bearing is contaminated with noise, Yang et al. [97] propose a method based on mutual information and sample entropy. The kurtosis value of the weak signal is increased by 3.2 times with minimum entropy deconvolution. In addition, the sample entropy is proved to have powerful effects on condition monitoring of bear by studying the field data of the wind turbine transmission system, as given in [98].

### 3.6. Application of Approximate Entropy on Bearing

The application of approximate entropy on bearing condition monitoring is given in Table 6. To identify the different health conditions of rotating machinery, a novel fault diagnosis method based on the modified multi-scale symbolic dynamic entropy and minimum redundancy maximum relevance is proposed [99]. The approximate entropy is used as a nonlinear feature parameter to measure the irregularity of vibration signal in the fault diagnosis of rotating machinery and the empirical mode decomposition method is used to improve the distinguishability of the approximate entropy values of different faults [100]. One kind of frequency-weighted energy operator and complementary ensemble empirical mode decomposition for bearing fault detection is proposed in [101], which overcomes the mode mixing and eliminates the residual contaminated with white noise. An et al. [102] propose one kind of bearing fault diagnosis method, which is based on the adaptive local iterative filtering and approximate entropy. It has been verified that the unsteady characteristics of a fault vibration signal from a wind turbine rolling bearing can be identified. In addition, approximate entropy can be used to detect cracks, which can effectively differentiate the occurrence of crack and misalignment, as given in [103].

### 3.7. Application of Fuzzy Entropy on Bearing

Some representative studies based on fuzzy entropy for bearing condition assessment are given in Table 7. A new rolling bearing fault diagnosis method based on the local characteristic-scale decomposition and the fuzzy entropy is proposed in [104]. The vibration signal of bearing is processed by local characteristic-scale decomposition and fuzzy entropy, which is used to extract fault features from the intrinsic scale components. The partial ensemble empirical mode decomposition and fuzzy entropy are used to solve the mode mixing problem existed in empirical mode decomposition [105]. Yang et al. [106] propose one kind of vibration signal de-noising method based on improved intrinsic timescale decomposition and extracted fuzzy entropy as the fault feature of the rolling bearing. Zheng et al. [49] propose a bearing fault diagnosis method based on multi-scale fuzzy entropy, Laplacian score, and variable predictive model-based class discrimination.

Compared with approximate entropy and sample entropy, multi-scale fuzzy entropy considers the dynamic nonlinearity, interaction and coupling effectiveness among different mechanical components. Therefore, it can provide more hidden information in different scales of the vibration signals. Zhao et al. [107] propose one kind of new feature extraction method based on ensemble empirical mode decomposition and multi-scale fuzzy entropy for bearing. Li et al. [108] improve the composite multiscale fuzzy entropy on reliability and stability and combine it with an infinite feature selection algorithm and support vector machine as an intelligent fault identification method. To measure the similarity of patterns between normal signals and tested signals of bearing, the cross-fuzzy entropy is proposed in [109]. A method of rolling bearing multi-fault diagnosis based on the fuzzy entropy and empirical mode decomposition, principal component analysis, and self-organizing map neural network is applied in [110]. This method solves the problem that using empirical mode decomposition in tandem with principal component analysis to extract fault features may lead to imprecise classification. In [111], the integrating empirical wavelet transform is used with fuzzy entropy for fault diagnosis of bearing.

Methods of combining fuzzy entropy theories with support vector machines are widely used for bearing fault diagnosis. Zhu et al. [112] propose a method based on adaptive local iterative filtering, modified fuzzy entropy and support vector machine. Liu et al. [113] improve the multiscale fuzzy entropy and combine it with the Laplacian support vector machine, which can achieve higher recognition rates and better robustness than multiscale fuzzy entropy algorithm. To improve the performance of multiscale fuzzy entropy for complexity measure of short data series, Zheng et al. [114] propose the Sigmoid-based refined composite multiscale fuzzy entropy. For the same problem, Zhu et al. [115] apply the time shift multiscale fuzzy entropy to the complexity analysis of data series. A new method for bearing fault diagnosis is based on this idea with the Laplacian support vector machine. 

### 3.8. Other Typical Entropy Theories Application on Bearing

There are many other typical entropy theories applied for condition monitoring of bearing, as given in Table 8. The main research objectives are fault diagnosis and prognostics of bearing.

The key to achieving near-zero downtime and maximum productivity is performance degradation. Zhu et al. [116] propose a method for performance degradation based on hierarchical entropy and general distance. Two specific steps are processed in this method. First, hierarchical entropy is employed to extract feature vectors. Secondly, Euclidean distance and cosine angle distance are combined as a degradation indicator. Similarly, Pan et al. [117] propose the spectral entropy as an index for performance degradation assessment of bearing. A method based on entropy changes at specific frequency is applied to predicting remaining cycle before maintenance [118], in which degradation feature is extracted from decomposed signals into frequency domain. The vibration intensity of bearing has both random non-stationary and long-range dependent characteristics when a weak fault occurs. Fractional Brownian motion combined with minimum entropy deconvolution is applied to evaluating the condition of bearing fault with a gradual process, as given in [119].

Some related researches on bearing fault diagnosis aim at achieving more accurate and more time-sensitive on feature extraction. Han et al. [120] propose a method based on ensemble empirical mode decomposition and cloud model characteristic entropy. The cloud model characteristic entropy has more advantages than traditional entropy complexity in parameter selection when solving uncertainty problems. Thus, it is defined as the eigenvalue of the reconstructed signals in this method. In [121], ensemble empirical mode decomposition is combined with improved frequency band entropy for bearing fault extraction. Zhang et al. [122] propose a method based on empirical mode decomposition, clear iterative interval threshold, and the kernel-based fuzzy c-means eigenvalue extraction. In this way, the problems of weak defect signals and large acoustic emission data in low-speed bearing condition monitoring are solved. Fine-sorted dispersion entropy combined with mutation sine cosine algorithm and particle swarm optimization optimized support vector machine is presented to diagnose faults of different sizes, locations and motor loads [123]. In [124], stationary wavelet packet Fourier entropy is used to extract fault features and kernel extreme learning is applied to dealing with these fault features, which can achieve better accuracy results than stationary wavelet packet permutation entropy and stationary wavelet packet dispersion entropy.

## 4. Condition Monitoring of Gear 

**Figure 4:**
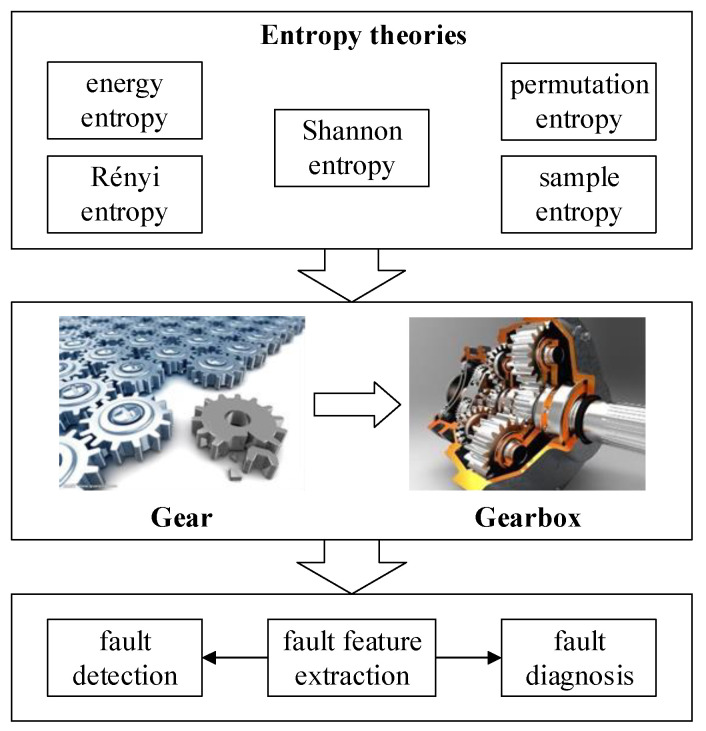
Related entropy theories application in condition monitoring of gear.

Compared with studies on bearing, the related researches on gear condition monitoring are not that much. As one kind of power transmission, the condition of gear decides whether the rotating machinery can work continuously and safely. In the practical application, gear is often equipped in the gearbox. Therefore, some studies have paid attention to the condition monitoring of gearbox. The related works in this category are illustrated in Figure 4.

### 4.1. Application of Shannon Entropy on Gear

Two representative studies using Shannon entropy on gear condition monitoring are given in Table 9. The fault detection of a gearbox with multi-component signals is full of difficulties caused by its complexity and instability. An adaptive redundant multiwavelet packet method for the compound-fault diagnosis is proposed, in which the minimum sum of normalized multifractal entropy is adopted as the optimization metric and the relative energy ratio of the characteristic frequency is utilized for automatically selecting the sensitive frequency bands [125]. The re-sampling technique and the continuous wavelet transform are fused to obtain the wavelet coefficients of the monitoring data. Based on the maximum energy to Shannon entropy ratio criteria, the optimal range of wavelet scales is selected and feature vectors are reduced [126].

### 4.2. Application of Energy Entropy on Gear

Three typical studies based on energy entropy for gear condition monitoring are illustrated in Table 10. When some faults occur in different parts of machinery, their features are dependent on each other. However, problems arise in this situation and the separation of features becomes complicated. To overcome these problems, Asr et al. [127] proposed a method that only individual fault features in training steps are applied instead of combining fault features as training data set. Empirical mode decomposition is used to decompose multi-component signals into internal mode functions. The appropriate internal mode functions for feature extraction are selected by using correlation coefficients. Shannon energy entropy and statistical features of the internal mode functions are extracted in the feature extraction step to realize fault detection.

The improved empirical mode decomposition can enhance the final effectiveness of empirical mode decomposition, which is used to decompose the signals to obtain intrinsic mode function [128]. Meanwhile, the improved empirical mode decomposition energy entropy which reflects the working state is extracted as the input of the support vector machine. The gear fault can be detected by comparing the energy distribution of the gear vibration signal. Hilbert–Huang transform can offer the energy–frequency–time distribution [129]. At the same time, Shannon entropy can be a useful criterion for analyzing and comparing probability distribution.

### 4.3. Application of Permutation Entropy on Gear

Two studies on gear condition monitoring using permutation entropy are given in Table 11. Mao et al. [130] present a joint fault diagnosis method which combines tensor nuclear norm canonical polyadic decomposition (TNNCPD) with multi-scale permutation entropy. TNNCPD is used to extract the low-rank component of the data, which describes the feature information of the measured signal. Fault conditions can be recognized by the feature vector which is calculated by the Multi-scale permutation entropy of the extracted feature information about different gear faults. Kuai et al. [131] propose a method based on permutation entropy of ensemble empirical mode decomposition with adaptive noise. The adaptive neuro-fuzzy inference method is adopted for diagnosing faults in the planetary gear. The complete ensemble empirical mode decomposition with adaptive noise decomposes the original signal into 6 intrinsic mode functions and residual components. Time complexity of intrinsic mode functions is reflected by permutation entropy to quantify fault features. The reason is that the intrinsic mode function contains the key characteristic information of planetary gear fault.

### 4.4. Other Typical Entropy Theories Application on Gear

Some other typical studies based on entropy theories for gear condition monitoring are shown in Table 12. To realize the diagnosis of gearboxes in presumably non-stationary and unknown operating conditions, the fusion of wavelet pack transform and Rényi entropy can be adopted [132]. The detailed analysis is mainly based on the probability density of the envelope of a sum of sinusoidal signals with random amplitude and phase. The authors also provide the conclusion that fault detection is possible without information about the operating conditions. Another effective method for planetary gear consists of two steps, including entropy feature fusion of dual-tree complex wavelet transform and optimized kernel Fisher discriminant analysis [133]. Zhang et al. [134] improve dual-tree complex wavelet transform and combine it with minimum entropy deconvolution to diagnose the composite fault of a gearbox. It extracts the outer ring fault at a frequency of 160 Hz, the gearbox fault with a characteristic frequency of 360 Hz and its double frequency of 720 Hz.

In [135], one kind of fault diagnosis method of planetary gear using the entropy feature fusion of ensemble empirical mode decomposition is proposed. The original feature set is composed of various entropy features of each intrinsic mode function. To address the insensitive features in the original feature set and the excessive feature dimension, kernel principal component analysis is utilized to process the original feature set. Also for fault diagnosis of planetary gear, a method based on fuzzy entropy of local mean decomposition (LMD) and adaptive neuro-fuzzy inference system (ANFIS) is proposed in [136]. Fuzzy entropy is used to reflect the complexity and irregularity of LMD. The optimal ANFIS mode is acquired and fuzzy inference rules are determined by defining the fuzzy entropy as the input of ANFIS model. Fault diagnosis of sun gear has always been a technical challenge because sun gear is in the center of a gearbox, which leads to the inconvenience of measuring. In [137], a fault diagnosis method based on continuous vibration separation (CVS) and minimum entropy deconvolution (MED) is proposed for solving this problem. In this method, CVS is utilized to overcome modulation effect caused by planetary movements, which can restrain noises and asynchronous components. MED is applied to enhancing fault induced impulses. Tang et al. [138] propose a method based on hierarchical instantaneous energy density dispersion entropy (HIEDDE) and dynamic time warping (DTW). HIEDDE is defined as the fault feature, which is calculated by the hierarchical dispersion entropy (HDE) algorithm. The instantaneous energy density signal, which is the computing objective for HDE, is acquired from singular spectrum decomposition and Hilbert transform. The DTW algorithm is employed to obtain the fault type. Similar with this motivation, a method based on the combining product function and multipoint optimal minimum entropy deconvolution adjusted (MOMEDA) is proposed in [139]. Specifically, this method consists of four parts. First, ensemble local mean decomposition and correlation coefficient method are utilized to get the series of product functions (PFs). Secondly, PFs without periodic impact are removed. Thirdly, PFs with the same period are recombined by the combined product function method. Finally, the fault feature is further extracted by MOMEDA.

## 5. Condition Monitoring of Other Rotating Machinery

**Figure 5:**
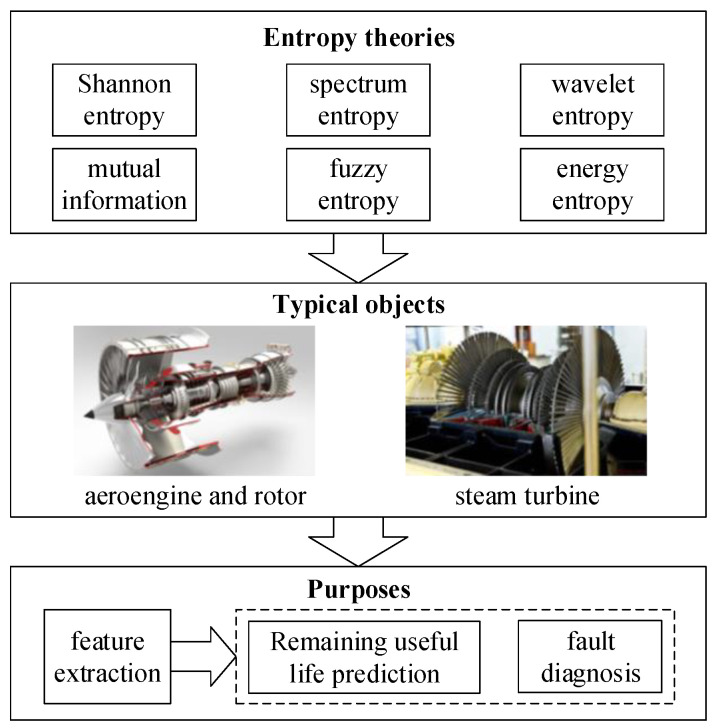
Condition monitoring of other typical rotating machinery with related entropy theories.

Besides bearing and gear, the related entropy theories have been applied in other kinds of rotating types of machinery. The related works are summarized in Figure 5.

### 5.1. Typical Entropy Theories Application on Fault Detection of Other Rotating Machinery

The applications of related entropy theories on other rotating machinery are summarized in Table 13. Rostaghi et al. [140] use the dispersion entropy to monitor the condition of rotating machinery because it has the advantage of quantifying the uncertainty of the signal. Experimental results show that dispersion entropy can characterize the state of rotating machinery more precise than permutation entropy and approximate entropy. In [141], the ambiguity measure, which is an entropy-like uncertainty measure in Dempster Shafer evidence theory, is applied to realizing fault evaluation of aircraft turbine rotor blade. To analyze the coupling characteristics of fault signals under the influence of complex and nonlinear interference signals, a method based on harmonic-assisted multivariate empirical mode decomposition and transfer entropy is proposed in [142]. The high-frequency harmonic-assisted multivariate empirical mode decomposition method is used to extract features of the mechanical transmission system and the signal is used to calculate the transfer entropy with noise. In addition, a method based on the improved AR-minimum entropy deconvolution and variational mode decomposition approach is proposed to extract the incipient single-fault and multi-fault from the nonlinear and non-stationary vibration signals with the strong background noise [143]. Shannon entropy is adopted to measure the uniformity of exhaust temperature and vibration data, which can provide useful information for fault diagnosis [144]. 

### 5.2. Typical Entropy Theories Application on Fault Diagnosis of Other Rotating Machinery

Some typical studies on other rotating machinery are given in Table 14. The vibration data of wind turbines are nonlinear and non-stationary. To effectively diagnose faults for wind turbines, Chen et al. [145] propose a method based on variational mode decomposition (VMD) and energy entropy. VMD can reflect signal components more accurately than empirical mode decomposition with less modal decomposition layer. Similar to this method, a method based on manifold learning and Shannon wavelet support vector machine is used in [146]. The Shannon wavelet support vector machine is applied to dealing with the low-dimensional eigenvectors which are compressed from high-dimensional feature. In addition, Xiao et al. [147] propose a method based on dual-tree complex wavelet transform energy entropy to classify the misalignment of the transmission system of the wind turbine. In order to solve the problem of insufficient sensor data from the working engine, information entropy and deep belief networks are used for gas turbine engine fault diagnosis in [148]. A method based on time-frequency entropy enhancement and boundary constraint assisted relative gray relational grade are applied for fault diagnosis for an autonomous underwater vehicle, as given in [149]. Shannon entropy is used to measure the uniformity of exhaust temperature and vibration data in [144,150]. The fusion of process power spectrum entropy and support vector is proposed to realize four typical faults, including rotor imbalance, shaft misalignment, rotor-stator rubbing, and pedestal looseness [151]. Wavelet correlation feature scale entropy and fuzzy support vector machine are used to analyze the whole-body vibration signal of aero-engine [152]. For the fault diagnosis of diesel engines, the energy entropy is utilized as a fault feature which is the input of complete ensemble intrinsic time-scale decomposition [153]. The basis of the vague cross-entropy is applied to realizing the fault diagnosis of turbine. Not only the main fault types of turbine can be diagnosed, but also the future trend and multi-fault analysis can be predicted [154]. 

A nonlinear projection noise reduction method based on smooth local subspace projection method and permutation entropy is proposed in [155]. Permutation entropy is applied in the detection of time sequence randomness and dynamic mutation behavior. Wang et al. [156] improve the multi-scale permutation entropy, which is named as an optimized multi-scale permutation and uses it to extract fault features. Multiscale symbolic dynamic entropy (MSDE) has been used for fault diagnosis of rotating machinery, which has merits of high computational efficiency and robustness to noise, as given in [157]. Composite multiscale symbolic dynamic entropy is proposed to address the inadequacy that the variance of the MSDE values increase as the length of data series becomes shorter using multiscale analysis. Another fault diagnosis method is the entropy-based feature extraction and support vector machine optimized by a chaos quantum sine cosine algorithm [158]. Specifically, this method consists of three parts. First, VMD is utilized to decompose the vibration signals into sets of components. Secondly, the permutation entropy value of each component is calculated to constitute the feature vector. Finally, support vector machine is optimized and the fault pattern is recognized. In [159], singular spectrum entropy, power spectrum entropy, and approximate entropy are extracted in vibration signals by Shannon entropy, and the feature fusion model is constructed to classify and diagnose the fault signals.

### 5.3. Typical Entropy Theories Application on Fault Prognostics of Other Rotating Machinery

Prognostics is a difficult problem for rotating machinery. One reason is that the sensing data are often contaminated with noise. As illustrated in the aforementioned studies, some entropy theories and signal processing methods can be utilized to implement feature extraction. Another reason is that the sensing data do not have a monotonous trend that is useful information for prognostics. For example, vibration data are hard to extract this feature. Some studies have pay attention to utilize entropy theories to realize this objective. One strategy is to select the sensors based on entropy [31], which can provide the quantity of information. Another strategy is to achieve quantitative description of monotonous trends, which can be used to select appropriate sensors for implementing prognostics [33]. Mutual information, which is based on the Shannon entropy, is employed to measure the relationship among multiple sensors data. Then, the appropriate sensors data can be selected to predict the condition of aircraft engine and aircraft auxiliary power unit [160].

In addition, the utilization and reliability of sensor data have a direct influence on the condition monitoring result of objectives. In [161,162], the anomaly detection of sensing data is studied to avoid its influence on the condition assessment of the system. If the sensor itself becomes anomalous, its data can be recovered to enhance the monitoring result [163,164]. If the sensor anomaly detection and data recovery are implemented together, it is especially useful for the on-line application [165]. Though the accuracy and stability of monitoring results are reduced, it provides a positive solution to keep on-line condition monitoring run continuously.

## 6. Case Study

**Figure 6:**
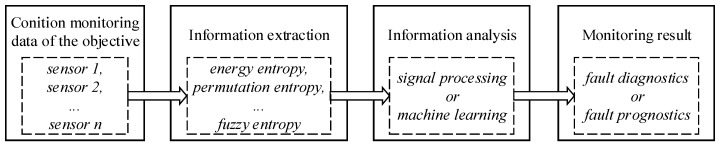
The process of related entropy theories applied to condition monitoring of rotating machinery.

In Section 3, Section 4 and Section 5, the applications of related entropy theories (energy entropy, permutation entropy, etc.) on condition monitoring of bearing, gear, and other rotating machinery have been extensively reviewed. The typical process using entropy theories includes three steps, as summarized in Figure 6. First, related entropy theories are used to mine the useful information contained in the condition monitoring raw data of the objective. The condition monitoring data are usually collected by sensors. In general, feature extraction or feature enhancement is realized by utilizing such transformation due to the insufficient feature representation capability of the raw sensor data. Then, by utilizing signal processing or machine learning methods, the extracted information can be analyzed appropriately. Finally, fault diagnosis or fault prognostics can be achieved. Figure 6 indicates a general process of utilizing related entropy theories to assess the condition of rotating machinery. In this section, we follow these steps to implement the case study, which is expected to provide a guideline for researchers, especially for new researchers who intend to contribute to this field.

The studies on fault diagnosis account for over 90% of reviewed articles. One reason may be that the fault information contained in the condition monitoring data of fault objective are fixed. For example, the collected data of crack bearing contain the crack fault information. If the proposed method can mine the fault information more effectively, the new contribution to fault diagnosis is achieved. In contrast, fault prognostics needs to extract the health variation information of the objective, which is not easy to be realized. One typical research direction is to predict remaining useful life (RUL) of the machine. In general, the degradation information is basically required to implement RUL prediction [33], which usually has a monotonous trend. In fact, the monotonous trend among sensor data can be depicted by the relationship among several adjacent data to some degree, which has a certain match with the definition of permutation entropy. For a sequence of data, if the next data is always larger or smaller than the current data (one kind of permutation feature), this data sequence has a strictly monotonous trend. Therefore, permutation entropy is selected to implement the following case study. In addition, the application of other entropy theories is also through this similar analysis process.

For selecting the condition monitoring data set, the challenge data of the 2008 Prognostics and Health Manage Conference are utilized. This data set is about the condition monitoring of the gas turbine engine. The details of this data set can be found in [166]. To be specific, there are 21-dimension monitoring data collected from sensors deployed in the engine. Among these data, some sensors data can provide useful information for implementing RUL prediction (i.e., the data with the feature of monotonous trend). The traditional method of selecting sensors data for prognostics method is to observe sensors data which have a monotonous trend. These data can represent the degradation of the engine to a large degree. In contrast, the selection of appropriate sensors data with quantitative metrics is difficult. Related entropy theories are based on the analysis of numerical calculation that can provide quantitative information about condition monitoring data. Then, the achieved information can be used to carry out prognostics. Therefore, the flow of applying different entropy theories on condition monitoring of the objective is similar.

In this case study, we carry out the comparison between the observation method and the permutation entropy selection method for selecting data for the input of prognostics. The 21 sensors are named from 1# to 21#. The utilized sensors in [167] are used to implement the comparison study, which includes 2#, 4#, 7#, 8#, 11#, 12#, and 15#. According to the study in [33], the selection 7 sensors data based on permutation entropy are 3#, 4#, 8#, 9#, 14#, 15#, and 17#. These sensors data are utilized as the input of RUL prediction, which is one of the most important technologies in the domain of prognostics. The details of comparison experiments are illustrated in three terms.

(1) These two groups of sensors data are used as the input of Gaussian process regression (GPR) to realize the RUL prediction of the gas turbine engine. Results will be compared and analyzed to evaluate the effectiveness of the permutation entropy method.

(2) Another data-driven method will also be utilized to achieve the RUL prediction of the gas turbine engine, which is the relevance vector machine (RVM). The prediction results of two groups of sensors data will also be compared and analyzed. 

(3) To evaluate RUL prediction results, mean absolute error (MAE) and root mean square error (RMSE) are used to measure the precision and stability, respectively. Smaller numerical values represent better prediction results.

**Figure 7:**
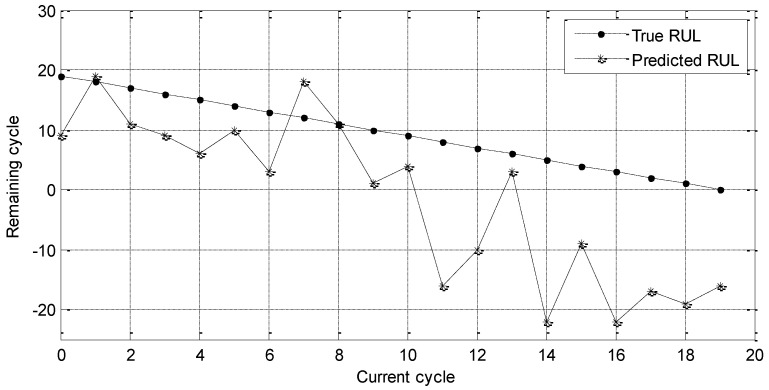
RUL prediction results of the selected sensors by observing method.

The details of GPR and RVM will not be given in this review. They belong to the category of the regression method and have been widely used for prediction applications. We first implement RUL prediction experiment with sensors 2#, 4#, 7#, 8#, 11#, 12#, and 15#. Experimental results are shown in Figure 7.

In Figure 7, the dotted line refers to the true RUL value and the star curve represents the predicted RUL value. After 10 cycles, the prediction RUL deviates the true RUL seriously. To depict the prediction results, MAE and RMSE values are calculated as follows.

MAE = 11.55 cycle

RMSE = 14.03 cycle

**Figure 8:**
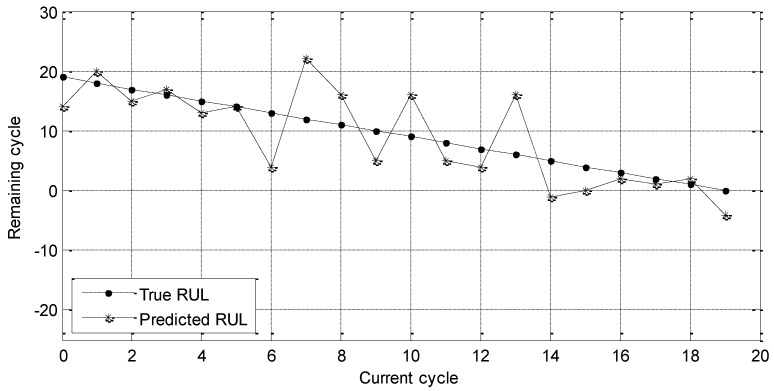
RUL prediction results of the selected sensors by permutation entropy.

Then, the RUL prediction experiment with sensors 3#, 4#, 8#, 9#, 14#, 15#, and 17# is carried out. Experimental results are shown in Figure 8.

The dotted line and star curve in Figure 8 have the same meaning as those in Figure 7. By observing these two figures, it can be seen that the predicted RUL in Figure 8 is close to the true RUL, which means that these experimental results are better. The corresponding MAE and RMSE are given as follows.

MAE = 4.05 cycle

RMSE = 5.03 cycle

By using the permutation entropy, MAE is improved from the 11.55 cycle to the 4.05 cycle and RMSE is improved from the 14.03 cycle to the 5.03 cycle. Therefore, the advantage of permutation method is evaluated. Then, RVM is used to implement the evaluation experiments further. Two kinds of experimental results are summarized in Table 15.

In Table 15, RUL prediction results of permutation entropy using RVM are also better than those of the observing method. MAE is improved from the 11.60 cycle to the 5.32 cycle and RMSE is improved from the 12.42 cycle to the 6.80 cycle. Due to the difference between GPR and RVM, the prediction results cannot be exactly same. The advantage of permutation method has been verified. Traditional researches focus on how to improve the performance of the utilized method. For example, the parameters optimization always attracts the attention of many researchers. Experimental results of this case study provide another way about how to improve the prediction results. Other kinds of entropy theories may have a similar ability to permutation ability.

## 7. Summary and Discussion

Based on the aforementioned studies, we can find that the related entropy theories have been widely applied in the domain of condition monitoring of rotating machinery. We try our best to review the most typical works in this domain. However, it may be inevitable that we have omitted some valuable works. By summarizing the reviewed works in the above sections, some issues should be still paid attention, as given as follows.

(1) Research on multiple dimension data analysis using related entropy theories should be considered. Most of the existing works focus on analyzing the vibration data. However, there are still some condition monitoring data which are not utilized enough to realize the condition assessment of rotating machinery.

(2) More complex rotating machinery should be studied by the related entropy theories. For example, more than five-level transmission of gearbox is applying more and more. However, the related condition monitoring works have not been reported. These gearboxes are usually utilized in some vital objects and the fault may bring catastrophic results.

(3) For the online application, the computation efficiency of entropy theories should be improved. In general, entropy theories are combined with other signal processing or machine learning methods. More computation sources are required. If the complex computation is not solved, it is hard to utilize the advantage of entropy theories for online application.

## Figures and Tables

**Table 1:** Applications of Shannon entropy in condition monitoring of bearing.

Index	Authors	Methodologies
1	Jiang et al. [50]	singular value decomposition + Shannon entropy
2	Kankar et al. [51]	support vector machine - learning vector quantization - self-organizing maps + Shannon entropy
3	Hemmati et al. [52]	wavelet packet transform + Shannon entropy
4	Reddy et al. [53]	empirical mode decomposition + Shannon entropy
5	Leite et al. [54]	Shannon entropy

**Table 2:** Applications of energy entropy in condition monitoring of bearing.

Index	Authors	Methodologies
1	Qin et al. [38]	ensemble empirical mode decomposition + energy entropy
2	Su et al. [55]	empirical mode decomposition + energy entropy
3	Jing et al. [56]	wavelet transform + energy entropy
4	Wan et al. [57]	Teager energy entropy ratio of wavelet packet transform
5	Yao et al. [58]	wavelet packet energy entropy + local outlier factor algorithm
6	Dong et al. [59]	local mean decomposition + energy entropy
7	Ao et al. [60]	local characteristic-scale decomposition + energy entropy
8	Kankar et al. [61]	energy to Shannon entropy ratio + Shannon entropy
9	Pang et al. [62]	characteristic frequency band energy entropy + support vector machine
10	Jiang et al. [63]	energy entropy theory + hybrid ensemble auto-encoder

**Table 3:** Applications of permutation entropy in condition monitoring of bearing.

Index	Authors	Methodologies
1	Li et al. [65]	local mean decomposition + multiscale permutation entropy
2	An et al. [64]	variational mode decomposition + permutation entropy
3	Liu et al. [66]	variational mode decomposition + multiscale permutation entropy
4	Shi et al. [67]	local mean decomposition + permutation entropy
5	Xue et al. [68]	ensemble empirical mode decomposition + permutation entropy
6	Yao et al. [69]	ensemble empirical mode decomposition + multiscale permutation entropy
7	Zhang et al. [70]	singular value decomposition + permutation entropy
8	Wang et al. [71]	wavelet packet transform + permutation entropy
9	Zhao et al. [72]	wavelet packet decomposition + multiscale permutation entropy
10	Fu et al. [73]	variational mode decomposition + permutation entropy
11	Yan et al. [74]	improved variational mode decomposition + instantaneous energy distribution-permutation entropy
12	Yasir et al. [75]	multi-scale permutation entropy
13	Tian et al. [76]	permutation entropy + manifold-based dynamic time warping
14	Lv et al. [77]	permutation entropy
15	Zheng et al. [78]	support vector machine + multiscale permutation entropy
16	Xu et al. [79]	compound multiscale permutation entropy + particle swarm optimization–support vector machine
17	Li et al. [80]	improved multiscale permutation + least squares support vector machine
18	Huo et al. [81]	permutation entropy + Laplacian score + support vector machine
19	Li et al. [82]	permutation entropy + improved support vector machine
20	Dong et al. [83]	time-shift multi-scale weighted permutation entropy + gray wolf optimized support vector machine
21	Zhou et al. [84]	weighted permutation entropy + improved support vector machine ensemble classifier
22	Tiwari et al. [85]	adaptive neuro fuzzy classifier + multiscale permutation entropy
23	Yi et al. [86]	tensor-based singular spectrum algorithm + permutation entropy
24	Zhang et al. [87]	feature space reconstruction + multiscale permutation entropy
25	Zheng et al. [88]	multi-scale weighted permutation entropy + extreme learning machine
26	Xue et al. [89]	two-step scheme based on permutation entropy + random forest

**Table 4:** Applications of Rényi entropy in condition monitoring of bearing.

Index	Authors	Methodologies
1	Bokoski et al. [90]	Rényi entropy + Gaussian process model
2	Tao et al. [91]	Rényi entropy
3	Singh et al. [92]	Rényi entropy + ensemble empirical mode decomposition

**Table 5:** Applications of sample entropy in condition monitoring of bearing.

Index	Authors	Methodologies
1	Liang et al. [93]	ensemble empirical mode decomposition + sample entropy
2	Zhang et al. [94]	lifting wavelet package transform + sample entropy
3	Seera et al. [95]	power spectrum + sample entropy
4	Han et al. [96]	local mean decomposition + sample entropy +energy ratio
5	Yang et al. [97]	mutual information + sample entropy
6	Ni et al. [98]	sample entropy

**Table 6:** Applications of approximate entropy in condition monitoring of bearing.

Index	Authors	Methodologies
1	Li et al. [99]	variational mode decomposition + approximate entropy
2	He et al. [100]	empirical mode decomposition + approximate entropy
3	Imaouchen et al. [101]	complete ensemble empirical mode decomposition + approximate entropy
4	An et al. [102]	adaptive local iterative filtering + approximate entropy
5	Sampio et al. [103]	approximated entropy

**Table 7:** Applications of fuzzy entropy in condition monitoring of bearing.

Index	Authors	Methodologies
1	Zheng et al. [104]	local characteristic-scale decomposition + fuzzy entropy
2	Zheng [105]	partially ensemble empirical mode decomposition + fuzzy entropy
3	Yang et al. [106]	intrinsic timescale decomposition + fuzzy entropy
4	Zheng et al. [49]	variable predictive model based class discriminate + multiscale fuzzy entropy
5	Zhao et al. [107]	ensemble empirical mode decomposition + multiscale fuzzy entropy
6	Li et al. [108]	composite multiscale fuzzy entropy
7	Zhu et al. [109]	cross-fuzzy entropy
8	Zair et al. [110]	fuzzy entropy of empirical mode decomposition + principal component analysis + self-organizing map neural network
9	Deng et al. [111]	integrating empirical wavelet transform + fuzzy entropy
10	Zhu et al. [112]	adaptive local iterative filtering + modified fuzzy entropy + support vector machine
11	Liu et al. [113]	composite interpolation-based multiscale fuzzy entropy+ Laplacian support vector machine
12	Zheng et al. [114]	sigmoid-based refined composite multiscale fuzzy entropy
13	Zhu et al. [115]	multiscale fuzzy entropy + Laplacian support vector machine

**Table 8:** Applications of other entropy theories in condition monitoring of bearing.

Index	Authors		Methodologies
1	Zhu et al. [116]	hierarchical entropy + general distance	
2	Pan et al. [117]	spectral entropy	
3	An et al. [118]	entropy changes at specific frequencies	
4	Song et al. [119]	fractional Brownian motion + minimum entropy deconvolution	
5	Han et al. [120]	ensemble empirical mode decomposition + cloud model characteristic entropy	
6	Li et al. [121]	ensemble empirical mode decomposition + improved frequency band entropy	
7	Zhang et al. [122]	empirical mode decomposition + clear iterative interval threshold + kernel-based fuzzy c-means eigenvalue extraction	
8	Fu et al. [123]	fine-sorted dispersion entropy + mutation sine cosine algorithm + particle swarm optimization optimized support vector machine	
9	Rodriguez et al. [124]	wavelet packet Fourier entropy + kernel extreme learning	

**Table 9:** Applications of Shannon entropy in condition monitoring of gear.

Index	Authors	Methodologies
1	He et al. [125]	adaptive redundant multiwavelet packet + Shannon entropy
2	Bafroui et al. [126]	continuous wavelet transform + Shannon entropy

**Table 10:** Applications of energy entropy in condition monitoring of gear.

Index	Authors	Methodologies
1	Asr et al. [127]	empirical mode decomposition + energy entropy
2	Xiao et al. [128]	improved empirical mode decomposition + energy entropy
3	Yu et al. [129]	empirical mode decomposition + energy entropy

**Table 11:** Applications of Permutation entropy in condition monitoring of gear.

Index	Authors	Methodologies
1	Mao et al. [130]	tensor nuclear norm canonical polyadic decomposition + multi-scale permutation entropy
2	Kuai et al. [131]	complete ensemble empirical mode decomposition + permutation entropy

**Table 12:** Applications of other entropy theories in condition monitoring of gear.

Index	Authors	Methodologies
1	Bokoski and Jurii [132]	wavelet packet transform + Rényi entropy
2	Chen et al. [133]	entropy feature fusion of dual-tree complex wavelet transform + optimized kernel Fisher discriminant analysis
3	Zhang et al. [134]	minimum entropy deconvolution + improved dual-tree complex wavelet transform
4	Cheng et al. [135]	ensemble empirical mode decomposition + sample entropy
5	Chen et al. [136]	fuzzy entropy
6	Zhang et al. [137]	continuous vibration separation + minimum entropy deconvolution
7	Tang et al. [138]	hierarchical Instantaneous energy density dispersion entropy + dynamic time warping
8	Cai et al. [139]	combining product function + multipoint optimal minimum entropy deconvolution adjusted

**Table 13:** Applications of the related entropy theories in condition monitoring of other rotating machinery.

Index	Authors	Methodologies
1	Rostaghi et al. [140]	dispersion entropy
2	Zhou et al. [141]	entropy-like measure
3	Wu et al. [142]	harmonic-assisted multivariate empirical mode decomposition + transfer entropy
4	Li et al. [143]	improved AR-minimum entropy deconvolution + variational mode decomposition approach
5	Wang et al. [144]	Shannon entropy

**Table 14:** Applications of the related entropy theories in fault diagnosis of other rotating machinery.

Index	Authors	Methodologies
1	Wang et al. [144]	Shannon entropy
2	Chen et al. [145]	variational mode decomposition + energy entropy
3	Tang et al. [146]	manifold learning + Shannon wavelet support vector machine
4	Xiao et al. [147]	dual-tree complex wavelet transform + energy entropy
5	Feng et al. [148]	information entropy + deep belief networks
6	Yin et al. [149]	time-frequency entropy enhancement + boundary constraint assisted relative gray relational grade
7	Chen et al. [150]	ensemble multiwavelet + Shannon entropy
8	Fei et al. [151]	support vector machine + process power spectrum entropy
9	Fei and Bai [152]	fuzzy support vector machine + wavelet entropy
10	Zhang and Liu [153]	ensemble intrinsic time-scale decomposition + energy entropy
11	Ye [154]	fuzzy cross-entropy
12	Fu et al. [158]	entropy-based feature extraction + support vector machine optimized by a chaos quantum sine cosine algorithm
13	Li et al. [157]	multi-scale symbolic dynamic entropy + improved support vector machine based on binary tree
14	Wang et al. [156]	optimized multi-scale permutation entropy
15	Xiao et al. [155]	smooth local subspace projection method + permutation entropy
16	Jiang et al. [159]	Shannon entropy + a probabilistic neural network

**Table 15:** Summary of evaluation experiments.

Prediction Method	Sensors Selection Method	MAE (Cycle)	RMSE (Cycle)
GPR	observing method	4.05	5.03
	permutation entropy	11.55	14.03
RVM	observing method	5.36	6.80
	permutation entropy	11.60	12.42
